# Development, Insults and Predisposing Factors of the Brain’s Predictive Coding System to Chronic Perceptual Disorders—A Life-Course Examination

**DOI:** 10.3390/brainsci14010086

**Published:** 2024-01-16

**Authors:** Anusha Yasoda-Mohan, Sven Vanneste

**Affiliations:** 1 Global Brain Health Institute, Trinity College Dublin, D02 R123 Dublin, Ireland; anusha.mohan@gbhi.org; 2 Trinity College Institute for Neuroscience, Trinity College Dublin, D02 R123 Dublin, Ireland; 3 Lab for Clinical & Integrative Neuroscience, School of Psychology, Trinity College Dublin, D02 R123 Dublin, Ireland

**Keywords:** risk factors, mis-match negativity, P300, EEG, neurotransmitters, prediction error

## Abstract

The predictive coding theory is currently widely accepted as the theoretical basis of perception and chronic perceptual disorders are explained as the maladaptive compensation of the brain to a prediction error. Although this gives us a general framework to work with, it is still not clear who may be more susceptible and/or vulnerable to aberrations in this system. In this paper, we study changes in predictive coding through the lens of tinnitus and pain. We take a step back to understand how the predictive coding system develops from infancy, what are the different neural and bio markers that characterise this system in the acute, transition and chronic phases and what may be the factors that pose a risk to the aberration of this system. Through this paper, we aim to identify people who may be at a higher risk of developing chronic perceptual disorders as a reflection of aberrant predictive coding, thereby giving future studies more facets to incorporate in their investigation of early markers of tinnitus, pain and other disorders of predictive coding. We therefore view this paper to encourage the thinking behind the development of preclinical biomarkers to maladaptive predictive coding.

## 1. Introduction

Perception is hypothesised to be a controlled hallucination, where the brain attempts to make sense of the incoming information in different contexts. This is widely agreed to follow the predictive coding framework [1,2]. According to this framework, the brain is considered to maintain an internal model of the external environment [1,2]. The predictions of this internal model (or priors) are compared with the sensory input (or likelihood) to produce a prediction error [1,2]. The priors and likelihood are associated with a precision (weighting), which determines the precision of the prediction error. Precise (or large) prediction errors can change the internal model or the perceptual inference of the world [1,2].

Such a change in perceptual inference is observed in healthy young adults during the perception of different types of illusions [3,4]. It is also observed as a natural phenomenon when exposed to a harmful stimulus—i.e., perceiving a ringing in the ears (tinnitus) after leaving a loud concert owing to a temporary shift in hearing thresholds [5], or perceiving pain following tissue damage [6]. Both of these perceptions go away when the respective sensory receptors revert back to normal.

However, in the chronic state, tinnitus and pain are accompanied by a myriad of biopsychosocial challenges. From a predictive coding perspective, we can hypothesise phantom perception as a maladaptive perceptual inference to minimise a prediction error owing to the overweighting of the sensory input or prior [7,8]. Permanent sensory nerve damage (deafferentation) can lead to an overweighting of the sensory input [9]. If this is not reduced through top–down inhibitory connections [10,11], this noise is hypothesised to reach consciousness resulting in a phantom perception—tinnitus or pain—depending on the modality of deafferentation [7,12]. Both in the auditory and somatosensory domains, phantom perceptions may be produced even in the absence of a sensory deafferentation. Although less understood why, we may attribute this to an overweighting of priors [8] or a change in the context of processing the same input. This is seen in patients with chronic tinnitus [13,14] and psychosomatic pain [15] where stress, anxiety and other traumatic incidents play a significant role in the generation of the phantom percept.

Both these chronic perceptual disorders affect a vast majority of the population (up to 15% for chronic tinnitus [16] and up to 40% for chronic pain [17]). Furthermore, in the U.S alone, tinnitus and chronic pain, respectively, cost the government USD 3.3 billion [18] and between USD 560 and USD 635 billion [19] annually, thereby imposing a huge socio-economic burden. Therefore, there is an urgent need to not only invest in the treatment of these disorders, but also in the identification of markers and prevention strategies for subsets of the population who may be at a higher risk of developing a chronic condition.

In this perspective, we take a life-course approach to (i) track how the predictive coding system develops from infancy, use phantom perceptions as a model to understand (ii) what are the changes in the neural and biomarkers of the predictive coding system in the acute and chronic phase, (iii) what are the neural and biomarkers that may indicate the transition from an acute to chronic phase and, finally, (iv) to explore developmental, environmental and genetic risk factors that may affect the maladaptive compensation of prediction errors, thereby identifying subsets of the population who may be at a higher risk of developing chronic perceptual disorders.

To build this perspective, we reviewed the literature in terms of the neural and biomarkers of predictive coding. We also reviewed the tinnitus and chronic pain literature in the acute, transition and chronic phase from the perspective of predictive coding to best capture the manuscripts that cover these neural and biomarkers of predictive coding. To highlight the factors that could predispose someone to maladaptive changes in predictive coding, we reviewed the biopsychosocial models for tinnitus and chronic pain combined with our experience and discretion to capture as many factors as possible.

## 2. Development of the Predictive Coding System through the Life Course

Predictive learning is shown to be the most efficient form of learning even outside the neuroscience realm. In humans, the development of higher-level cognition is proposed to originate from the predictive learning of sensorimotor signals in two ways: (i) updating an immature prediction error to refine one’s own sensorimotor abilities and (ii) executing an action estimated by the predictor’s response to others’ actions [20]. In her paper, Yuki Nagai describes how the predictor in the brain learns from early sensory input, such as reflexes, to build the proprioceptive sense of self and that of others. She states that the prediction error generated by sensory/motor input from the self is more predictable than that from an external source which is less predictable. The brain constantly works on reducing this prediction error and updating the model and learning to expand its abilities to higher levels of cognition, such as hand–eye co-ordination and understanding social and emotional cues.

Empirically, changes to this predictive coding system can be measured using different neuroimaging techniques. Particularly, sensory oddball tasks probe different features of the predictive coding network and characterise them in different components of the neural response. Oddball paradigms usually consist of a standard stimulus that is randomly interspersed with a deviant stimulus which acts as a surprise [21]. One of the theories behind this is that the brain records a temporary memory trace of the standard (which can be hypothesised to be the prediction) and the difference between the response of the deviant and the standard is the prediction error [22]. Through the life-course, oddball paradigms produce early and late components in infants and adults that record changes in sensory and cognitive processes.

### 2.1. Neural Markers

#### 2.1.1. Mismatch Negativity

Mismatch negativity (MMN) is a fronto-central negativity occurring around 100–200 ms post-stimulus presentation [21] that is known to encode prediction errors. In infants, pitch [23,24], timing [25,26] and deviants in melodies [27] create mismatch responses showing a very early sign of encoding prediction errors. Furthermore, MMN components change after only a few hours of exposure, reflecting learning [28]. For a long time, it was debated that MMN responses represented neural adaptation patterns given that they were recorded in response to changes in stimulus characteristics [29]. However, more recent studies in both adult humans and monkeys show that MMNs generated by the omission of a stimulus in a sequence cannot be explained by neural adaptations and are a reliable marker to prediction errors [22,30].

Using functional near-infrared spectroscopy (fNIRS), the authors demonstrated that infants showed the activation of the visual cortex during the unexpected and not during expected omission of a visual stimulus showing the early presence of prediction errors in the visual cortex [31]. Further evidence for the presence of predictions in infants was given using a cross-modal cueing paradigm where infants formed associations between auditory and visual cues that depended on the prior knowledge acquired [32]. In this study, predicted events produced a higher amplitude than unpredicted events in the early components of the event response potential and vice-versa during the later components.

#### 2.1.2. Late Positive Potential

MMNs record sensory prediction errors. Changes in cognitive components and more complex processes such as the updating of sensory memory occur later in time. In infants, an increase in a late positive component between 700–1000 ms, called the positive slow wave (PSW) [32], is traditionally considered as a marker representing the updating of memory specifically in response to partially encoded infrequent stimuli [33,34]. The PSW is also considered an early precursor of the P300 component observed in adults in response to oddball paradigms [33,35]. In adults, the P300 component is further divided into P3a and P3b depending on the paradigm and the task. In an active oddball paradigm with a standard, a target (rare stimulus that is attended to) and a novelty (surprise) stimulus, the target produces a posterior P3b component usually originating from the parietal cortex and the novelty produces an anterior P3a component that originates in the anterior cingulate [36]. The P3b is elicited on violating one’s predictions by the current input calls for updating internal models. Although the P300 is usually recorded as the target and novelty stimulus, its credibility to be a marker for prediction error is gaining traction [14,22].

### 2.2. Molecular Markers

The different components of the predictive coding system are guided by different neurotransmitters in the brain. Although there is no clear evidence of a one-on-one relationship between the neurotransmitter and component of the predictive coding system, pharmacological interventions and advanced neuroimaging techniques explore their dependence. Different drugs affecting glutaminergic transmission have been shown to affect the MMN. Ketamine, an N-methyl-D-aspartate receptor (NMDAR) antagonist, reduces the amplitude and delays the peak latency of the MMN [37,38]. The theory is that MMN, whether explained using neural adaptation models or memory-based predictive coding, is influenced by the NMDAR receptor [37].

Unlike the MMN, there is much discussion surrounding the neurotransmitters influencing the P300. An early review talked about the influence of the different neurotransmitter systems on the P300 [39]. They claimed that the P300 potential was directly influenced by the glutaminergic system since they are a direct result of excitatory post-synaptic potentials and indirectly by the GABA-ergic system through their inhibitory influence on excitatory post-synaptic potentials. In a seminal paper by Polich, he states that the P3a may be driven by the dopaminergic system and the P3b by the noradrenergic system [36]. This is backed up pharmacological and neuromodulatory interventions, showing the possible role of dopamine and noradrenaline in encoding the late positive potential [40,41]. Specifically, using an active inference model, a recent study shows how prediction errors trigger noradrenaline activity in the locus coeruleus which subsequently alters the rate of learning about the environment [42]. At a systems level, the noradrenergic activity of the locus coeruleus has been correlated with the amplitude of P3b [43].

**Figure 1:**
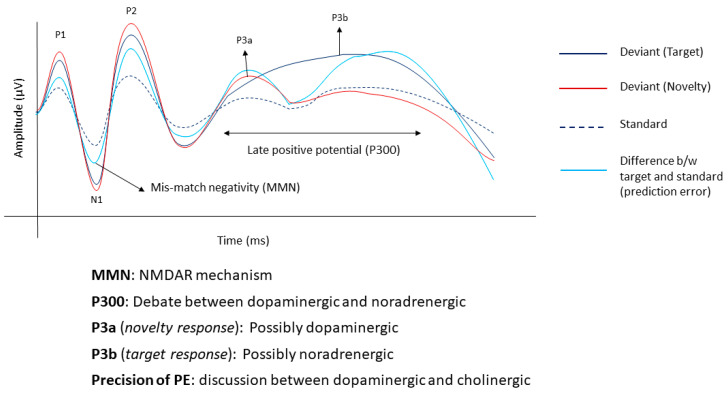
Summary of event-related potentials generated by a three-stimulus active oddball paradigm. The dashed black line shows the response to standard stimuli, the solid black line shows the response to the target (usually paid attention to) stimuli, the solid red line shows the response to a novel distractor stimulus and the solid blue line shows the difference in response to the standard and target.

However, there is still a lot of debate regarding the role of dopamine. Dopamine has been proposed to be involved in forming priors through associative learning [3]. Although there is some evidence, it is still not very clear if it is also involved in encoding the precision of the priors [44]. Some other authors suggest that dopamine is involved in the low-level precision weighted prediction error in trying to predict an outcome based on a given cue [45,46]. With respect to the precision of the prediction errors dominated by the likelihood, researchers associate this with the cholinergic activity in the basal forebrain [45]. This is backed up with the pharmacological modulation, neuroimaging and mathematical modelling of a system that constantly learns how to adapt to the changes in its environment [46,47]. Although the above studies try to somewhat explain a mutually exclusive relationship between different neurotransmitters and the different components of the predictive coding system, it is still a challenge to disentangle this relationship completely. A summary of the different neural and biomarkers of predictive coding is shown in Figure 1.

## 3. Changes in Predictive Coding in the Acute Phase of Phantom Perceptions

As introduced above, phantom perceptions may be a compensation of the brain to minimise a prediction error that has been brought on by a change in the incoming input or a change in the context in which the same input is presented. Therefore, it may be a natural phenomenon of the brain to perceive a phantom perception in an acute manner. In both acute tinnitus and pain, there are a myriad of temporary peripheral inconsistencies that potentially change the precision of the input. In the auditory domain, increased spontaneous emissions of the outer hair cells due to temporary damage following exposure to loud sounds and changing activity in the dorsal cochlear nucleus owing to stimulation through the somatosensory neurons, etc., may be some reasons [48]. These inconsistencies change the excitability of the peripheral nervous system which is relayed to the primary auditory cortex through the thalamus and perceived as phantom sound.

In the pain literature, the peripheral pathways are more comprehensively understood. The Aβ and Aδ fibres carry the “primary” pain signals, whereas the C fibres carry the “secondary” pain signals, both of which enter the spinal cord through the dorsal horn [49]. These are projected to the periaqueductal gray and on to the thalamus which relays this information to the somatosensory cortices through the lateral pain pathway and to the dorsal anterior cingulate cortex (dACC) and anterior insula (aI) through the medial pathways [49,50]. The lateral pathway records the sensory component of pain and the medial pathway records the emotional discomfort of pain [50]. Both pain and tinnitus are proposed to be modulated by a descending pathway—respectively, the first starting from the pregenual anterior cingulate cortex (pgACC) and going down through the periaqueductal gray [50], the other from the dlPFC and vmPFC to the thalamus [10,51]—that, from a predictive coding perspective, would reduce the precision of the incoming stimulus.

### 3.1. Neural Markers

Studies directly examining changes in predictive coding markers are sparse both in tinnitus and pain. However one study shows the increase in auditory evoked potential in the MMN time frame in the acute phase in both tinnitus [52] and pain [53]. Indirectly, ERPs examining the oddballs using nociceptive stimuli to generate acute pain showed that rare noxious stimuli produced a larger P300 amplitude compared to the standard, indicating an increase in the attention-related prediction error [54]. It was also seen that strong novel stimuli showed a bigger P300 response than weak novel stimuli when compared to the standard. Furthermore, this amplitude was enhanced with attention [54]. The role of acute pain on attention in relation to concurrent cognitive tasks showed that the amplitude of the late positive potential to a visual stimulus following a novel noxious stimulus was lower [54]. Unlike pain, tinnitus will have to be simulated through different proxies and illusions. Intermittent auditory ringing generated neural patterns that were similar to chronic tinnitus, showing a similar mechanism in the acute phase [4,55,56]. Further studies are need to understand brain dynamics in the acute phase to understand the transition to the chronic phase.

### 3.2. Molecular Markers

At the molecular level, the mechanisms that generate tinnitus and pain are slightly different, but overall involve an increase in the excitatory post-synaptic potential and increased spontaneous activity in neurons, which is interpreted as either perception. Following an acute injury, inflammatory mediators are released which act on G-coupled receptors to cascade a change in the threshold and kinetics of nociceptors to allow for a “soup” of molecules to bind with the nociceptors to regulate pain [57]. In tinnitus, acoustic trauma or ototoxic drugs could use one of the protein kinase pathways to increase opening calcium channels, or salicylate could increase the membrane conductance of the outer hair cells by either decreasing GABA or increasing NMDA to accelerate outer hair cell damage [58]. Salicylate and acoustic trauma can also change molecular events at the level of the auditory nerve synapse, cochlear efferents and at the level of the spiral ganglion [58] to cause an imbalance between excitatory and inhibitory dynamics to increase spontaneous activity in the peripheral nervous system. Theoretically, it would affect the input to the predictive coding system, but this has not been shown in the acute phase.

## 4. Changes in Predictive Coding in the Chronic Phase of Phantom Perceptions

In the chronic state, tinnitus and pain are more complex. In 2–5% of the population, the continuous awareness of the sound is associated with emotional distress, cognitive dysfunction and/or autonomic arousal leading to behavioural changes and functional disability. This is defined as tinnitus disorder [59]. Similarly, the International Association for the Study of Pain (IASP) defines chronic pain as “an unpleasant sensory and emotional experience associated with, or resembling that associated with actual or potential tissue damage” [60]. Tinnitus and pain are regarded as chronic when the perception lasts continuously for more than three months [61,62]. From a predictive coding perspective, in a chronic state, the brain learns that perceiving a phantom perception becomes the new norm, thereby changing the prediction of the brain to perceive a sound [9] (or pain).

### 4.1. Psychosocial Components

Both conditions in the chronic state are associated with sensory impairments that are specific to the auditory or somatosensory domains. About 90% of people with tinnitus have hearing loss that may or may not (hidden) be detected by an audiogram [63]. Although in chronic pain, the tissue damage itself may have resolved, there may be lasting changes in nerve sensitivity and inflammation [64]. Both conditions in the chronic state show changes in the central sensitisation [65,66] of the nervous system and a change in central gain [67,68] that arises because of peripheral nerve damage. Furthermore, increased sensitivity to sounds (hyperacusis) [69] and tactile stimuli (hyperalgesia [70]) is observed in patients with chronic tinnitus and pain, respectively.

The commonality between the two conditions is also seen in comorbid cognitive, mood and behavioural disorders. Dysfunctional attention in tinnitus is seen as slower reaction times on attention-oriented tasks and is mediated by tinnitus-related distress [71]. A similar observation has been made in patients with chronic pain and attention switching tasks [72]. Particularly, induced and chronic pain is shown to affect different aspects of attention [73]. Furthermore, in patients with chronic pain, an attentional bias is observed towards negative information on the Emotional Stroop task which is in turn related to higher levels of pain [74].

Emotionally, chronic tinnitus and pain are both accompanied by anxiety and depression. Among the 21.4 ± 0.69 million tinnitus sufferers in a cross sectional analysis of the 2007 Integrated Health Interview Series, 26.1% and 25% reported severe anxiety and depression, respectively, while only 9.2% and 9.1% of people without tinnitus reported the same [75]. Furthermore, people who reported tinnitus as a “big” or “very big” problem were significantly more likely to report anxiety and depression (OR = 5.7 and OR = 4.8) [75]. Similarly, an epidemiological survey of adults in the U.S. showed that those who reported chronic pain had an elevated severity of depression (PHQ-8 categories: none/minimal: 57.6%, mild: 22.3%, moderate: 11.4%, severe: 8.7%) and anxiety (GAD-7 categories: none/minimal: 66.4%, mild: 17.1%, moderate: 8.5%, severe: 8.0%) compared to those without chronic pain [depression (none/minimal: 87.6%, mild: 8.8%, moderate: 2.3%, severe: 1.2%; *p* < 0.001) and anxiety (none/minimal: 89.0%, mild: 7.5%, moderate: 2.1%, severe: 1.4%; *p* < 0.001)] [76]. Furthermore, 22.4% and 24.5% of chronic pain sufferers were taking medication for depression and anxiety, respectively, compared to 6.6% and 8.5% of those without chronic pain [76].

Finally, sleep disturbance is one of the most commonly noted behavioural effects of chronic tinnitus [77] and pain [78]. A cross-sectional assessment of the Mini Sleep Questionnaire (MSQ) in military personnel with tinnitus showed that MSQ scores were higher in 77% of patients compared to controls [79]. It was also noted that the regions involved in tinnitus were also the ones that were involved in REM sleep, posing a systems-level hypothesis of the relationship between the two [80]. A meta-analysis of sleep disturbances and sleep disorders in people with chronic pain showed that it was related with subjective and diagnosed sleep disorders [81].

### 4.2. Neural Markers

From a neurophysiological perspective, tinnitus and chronic pain show changes in widespread regions of the brain. Given that both disorders have a sensory and an affective component, they affect domain-specific and domain-general regions of the brain. The sensory component is encoded by an auditory network in tinnitus, consisting of primary and secondary auditory regions [82], and by the somatosensory network in chronic pain, consisting of the primary and somatosensory regions [83]. The broader domain-general network consists of regions that overlap with regions noted in the predictive coding framework of chronic tinnitus [9,84] and pain [85,86]. These are the dorsolateral prefrontal cortex (dlPFC), dorsal anterior cingulate cortex (dACC), ventromedial prefrontal cortex/pregenual anterior cingulate cortex (vmPFC/pgACC), subgenual anterior cingulate cortex (sgACC), posterior cingulate cortex (PCC), precuneus, inferior parietal cortex (IPC), anterior insula (aI), amygdala (AMG) and parahippocampus (PHC). These regions are divided among the default mode network [87] (DMN), the salience network [88] (SN) and a domain-general distress network [89,90] that is impacted both by chronic tinnitus and pain. Particularly, hyperactivity in the SN has been attributed to suffering related to pain [50] or tinnitus [91] and connectivity between the DMN and auditory networks has been attributed to the processing of internally directed perceptions and a change with hearing loss [87].

The predictive coding network was empirically tested in both chronic tinnitus and pain using oddball paradigms. In chronic pain, early [92,93,94,95] (MMN) and late [96,97] (P300) responses to auditory and visual oddball paradigms report a change in amplitude and latency. A reduced MMN amplitude is suggested to be correlated with the sensory component of the McGill Pain Questionnaire [92]. However, when subjected to somatosensory-evoked potentials, it seems like there is an increase in attention-related ERPs in the P300 timeframe showing an attentional bias to somatosensory stimuli in chronic pain patients [98,99].

In chronic tinnitus, changes to the predictive coding system were mostly recorded in the auditory domain with mixed results. While some studies show a decrease in the MMN amplitude [100,101,102], others show an increase in the MMN amplitude depending on the frequency and probability of the deviant [14,103]. Empirically, tinnitus becoming the new prediction has been shown as a reduction in the MMN amplitude to the tinnitus tone when presented as a deviant [53]. A meta-analysis of different ERP-based studies showed a reduction in the P300 amplitude [104]; however, in our recent study, we showed an increase in the P300 amplitude [14]. This difference may be attributed to the stimuli and the paradigm itself, where we used a local–global paradigm as opposed to the classic oddball paradigm, showing the hierarchical nature of the predictive coding system.

### 4.3. Molecular Markers

Tinnitus is often referred to as auditory pain and shares a lot of commonalities in terms of mechanisms [105]. Low glutamate and GABA levels were shown in a group with tinnitus [106] and chronic pain [107]. However, NMDA receptor activation by salicylate has shown to induce tinnitus [108], whereas NMDA receptor antagonists such as memantine and GABA agonist gabapentin [109] have shown to improve tinnitus. In patients with chronic pain, the level of glutamate and GABA changed with different types of pain [110]. Changes in GABA are also associated with changes in cortical inhibition and the disruption of the balance between excitation and inhibition [111]. Tinnitus is also considered to be maladaptive learning between the loudness of the sound and the distress because of the sound through the modulation of dopaminergic pathways [112]. Similarly, the dopaminergic modulation of different pain symptoms are also shown [113]. There are also changes in the predictive coding when transitioning from the acute to chronic phase.

## 5. Changes in Predictive Coding when Transitioning from Acute to Chronic Phase

### 5.1. Psychosocial Components

One of the most important differences between the acute and the chronic phases of change in perceptual disorders is the complex interplay of psychosocial factors in the chronic phase. This has been thoroughly examined in people with chronic pain rather than with tinnitus. A systematic review of the psychosocial aspects of the transition into chronic pain showed that the association with depression, fear avoidance, pain catastrophising, high traumatic exposure, emotional distress and the feelings of helplessness and hopelessness were key factors [114]. In tinnitus, preliminary evidence shows that between 6 weeks and 6 months of onset, the factors involved in the transition to a chronic condition include depression and anxiety levels, hyperacusis and the severity and frequency of hearing loss [115,116]. Another study showed that the subjective perception, moderate hearing loss and score on the Tinnitus Handicap Inventory were negatively associated with tinnitus chronicity [117]. However, it should be noted that this was a cross-sectional study.

### 5.2. Neural Markers

One of the hallmarks of chronic perceptual disorders is the central sensitisation and increase in central gain. This is seen as increased activity in the sensory regions of the predictive coding network, both in tinnitus and chronic pain. There are not many studies that show a direct relationship between the change in neural markers of predictive coding in the transition from the acute to chronic phase of perceptual disorders. In chronic pain, it is shown that the markers of prediction errors in the MMN timeframe are significantly larger in the acute phase compared to the chronic phase owing to this central sensitisation [53]. A similar observation is also made in tinnitus [52]. A cross-sectional comparison of changes in the activity and connectivity of predictive coding regions between acute and chronic tinnitus show increased activity in regions involved in predictive coding and connectivity between the PHC and PCC [118]. From a theoretical perspective, Sedley and colleagues proposed that in transition, tinnitus may involve the change of the prediction of the brain from perceiving silence in the absence of external stimuli to perceiving a ringing [9].

**Figure 2:**
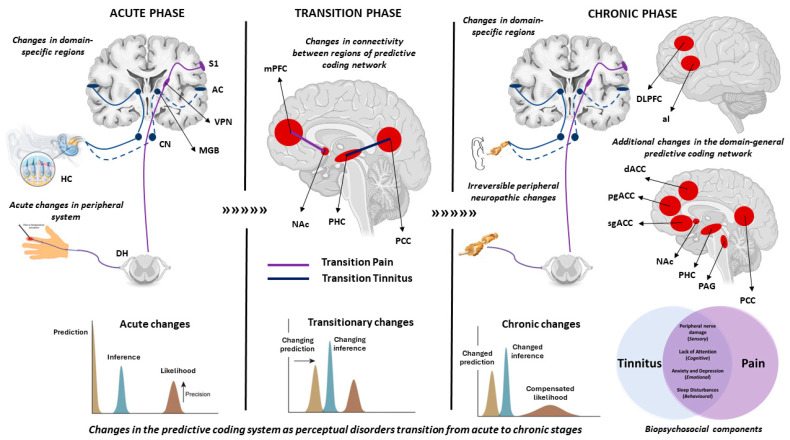
This figure summarises the neural markers and changes in the predictive coding system involved in the acute, transition and chronic phase of tinnitus and pain. In the acute phase, damage to the cochlear hair cells or an acute injury increase spontaneous activity in the peripheral system which is brought to consciousness by the domain-specific lateral pathway (blue = auditory, purple = somatosensory). Specific to predictive coding, there is an increase in the precision of the likelihood. In the transition phase, there are specific changes to connectivity between regions of the predictive coding network. In pain, the connectivity between mPFC and NAc predicts the transition from acute to chronic with over 80% accuracy. In tinnitus, a change in the connectivity between the PHC and PCC was noted in a cross-sectional study comparing acute and chronic patients. From a predictive coding perspective, there is a change in the prediction (from silence to perceiving a phantom) accompanied by a change in inference owing to a possible reinforcement learning facilitated by the limbic system. In the chronic phase, damaged peripheral nerves in the auditory and somatosensory systems cause a domain-specific central sensitisation, which together with a malfunctioning inhibitory pathway from the DLPFC or the pgACC for tinnitus and pain, respectively, bring the increased gain to consciousness. It is accompanied by a myriad of biopsychosocial components and a domain-general distress network (shown in red) whose regions also overlap with the predictive coding network. From a predictive coding perspective, the current prediction has now fully transitioned to expect the perception of the phantom as the new norm, thereby compensating for the increased precision of the likelihood and changing the inference as well. The abbreviations involved are HC = hair cells, DH = dorsal horn, S1 = primary somatosensory cortex, AC = primary auditory cortex, VPN = ventral posterior nucleus, MGB = medial geniculate body, CN = cochlear nucleus, mPFC = medial prefrontal cortex, NAc = nucleus accumbens, DLPFC = dorsolateral pre-frontal cortex, aI = anterior insula, dACC = dorsal anterior cingulate cortex, pgACC = pregenual anterior cingulate cortex, sgACC = subgenual anterior cingulate cortex, PHC = parahippocampal cortex, PCC = posterior cingulate cortex, PAG = periaqueductal gray.

A longitudinal follow-up of people who recovered or did not recover from subacute back pain over a period of one year showed that people who did not recover from the back pain (chronic state) showed a decrease in the grey matter volume in the insula, nucleus accumbens (NAc), striatum and the sensorimotor cortex [119]. Furthermore, they showed decreased connectivity between the hippocampus and medial prefrontal cortex (mPFC) compared to those who recovered [120]. Particularly, the connectivity between the mPFC and NAc determined the accuracy of the transition from the acute to chronic phase with >80% accuracy [121]. The NAc is part of the limbic system that is involved in the reinforcement learning of a positive or negative affect. This is in line with the hypothesis that chronic perceptual disorders may be a learned maladaptive association of the sensory component with the affective component leading to a shift in the brain’s prediction of the new norm [9,84]. The neural markers of the acute, chronic and transition phases are shown in Figure 2. The summary of these findings are summarised in Table 1.

### 5.3. Molecular Markers

Several markers that are neuronal, immune and glial are related to the chronification of pain through central sensitisation. One is through the recruitment of NMDA receptors and their activation in the dorsal horn through the increase in the post-synaptic glutamate receptor [122]. They increase calcium permeability causing long term plasticity and neuronal damage. The long-term maintenance of chronic pain involves the release of BDNF by modifying molecular transcriptional processes. It has been shown that the transcription of the genes c-Fos, cyclooxyfensae, neurokinin, TrkB and prodynorphin contribute to long-term central sensitisation [123]. On the other hand, BDNF released from microglia also has been shown in the development of chronic pain. The BDNF leads to a depolarising shift in the dorsal horn causing GABA-related signals to become excitatory, leading to a cascade of molecular shifts, ending in hyperalgesia [123]. The increase in excitation at the peripheral level is seen in the change between acute and chronic ERP markers where the amplitude in the MMN time frame is larger than in the chronic state. This indicates an increase in the peripheral sensitisation process leading to a more stable central sensitisation pattern. From our search of the literature, there are not a lot of studies that look into which molecular markers are involved in the transition from acute to chronic tinnitus. This presents a gap in the literature that can filled by future studies.

## 6. Factors Predisposing Children and Adults through the Life-Course to Maladaptive Changes to Predictive Coding and Perceptual Disorders

### 6.1. Developmental Insults

Infants are exposed to several kinds of insults pre- and postnatally that may directly or indirectly affect the development of the predictive coding system. In utero, foetuses exposed to drugs consumed orally or administered intravenously by pregnant mothers are born with withdrawal symptoms to the drugs called Foetal Alcohol Syndrome (FAS) or Neonatal Abstinence Syndrome (NAS). Children born with FAS experience physical birth deformations, growth retardation, facial dysmorphism, cognitive delay and behavioural abnormalities [124]. Studies show that FAS can also influence the morphology of the brain, subcortical structures, such as the hippocampus, olfactory bulb, cerebellum, pituitary and septal regions, and the ventricles, depending on the gestational time of consumption [125,126,127,128]. Particularly, the hippocampus and the cerebellum are key regions of the predictive coding system. NAS manifests as poor feeding, gastrointestinal disorders, abnormal sleeping patterns, jitteriness, tremors and seizures [129]. Studies with small samples of infants have shown FAS-related changes in the brain using EEG and MRI showing epileptic-type activity and changes in brain volume, specifically in the basal ganglia [130,131] which is also an important brain structure in the predictive coding system. Newborns are also exposed to other kinds of insults such as hydrocephalus, i.e., where there is a build-up of cerebrospinal fluid, and cerebral palsy (CP), a movement disorder that also affects co-ordination, swallowing, speaking and is accompanied by muscle stiffness weakness and tremors, etc.

Changes in the brain structure because of any of the above reasons, including pre-natal stress, can cause developmental delays due to the reduced ability of sensory-motor integration, which is the key step to cognitive development [132]. Furthermore, research suggests that more than half of the babies who are born pre-term (<=30 weeks) suffer from sensory processing disorders (SPD), which is a reliable precursor to developing developmental delays [133]. This is backed up by another systematic review of articles looking into SPD in children from birth—three born pre-term—showing sensory over-reactivity (82% of findings positive) in these children [134]. SPD is characterised by hyper-responsiveness, hypo-responsiveness or craving to sensory stimuli occurring in 5% of children in the general population and in 40–80% of the population with developmental disorders [135]. Furthermore, the severity of SPD in children predicts the severity of SPD in adulthood and whether or not these subjects will have high emotional dysregulation [136], which is suggested to be an important factor in the development and maintenance of chronic pain [137]. A detailed study using different sensory stimuli showed that children with SPD perceive more pain that lasts longer [138]. Although not as extensively researched in tinnitus, we know that tinnitus is sometimes accompanied by hyperacusis—or increased sensitivity to auditory stimuli—which is a subset of SPD.

### 6.2. Environmental Factors

The environment we are exposed to is constantly changing throughout our lifetime. From a predictive coding perspective, this changing environment plays a major role in shaping us into who we are, influences our belief systems and is paramount in forming associations. A meta-analysis showed a moderate risk of developing chronic pain in people with low and medium socioeconomic statuses [139]. This could be because one’s socioeconomic status determines the kind of obstetric care mothers receive during pregnancy, the nutritional value of the food infants receive during gestation, etc., which are important in determining the health of the new-born and are, in turn, key in the development of the sensory-motor system and cognition. An earlier study determined that infants with a lower birth weight and gestational age were more affected by birth-related complications than their peers with a normal birth weight and normal gestational age [140]. Furthermore, there is sufficient research stating the relationship between pre-term birth and SPD [133,134].

The next major factor is the health of the family environment. Several studies have shown the relationship between chronic pain and a history of physical and sexual abuse [141,142] and emotional neglect [143]. A meta-analysis examining the relationship between sexual abuse and a lifetime diagnosis of somatic disorders shows a significant risk of developing chronic pain (OR = 2.20; 95% CI, 1.54–3.15, I^2^ = 82%), particularly chronic pelvic pain (OR, 2.73; 95% CI, 1.73–4.30, I^2^ = 40%) [144]. Not only adverse childhood trauma, such as physical and sexual abuse, but also dysfunctional family dynamics can affect the neurodevelopment of a child. Although children coming from divorced parents are less likely to have gone through physical abuse, they are more likely to be diagnosed with ADHD [145]. Furthermore, there remains a strong association between early inpatient psychiatric hospitalisation and the development of mental illness later in their lives [145]. Children with a traumatic childhood background have also shown to have an increased association with dysfunctional sensory processing [146] which, as stated, is a risk factor for developing chronic pain and often observed with tinnitus.

In addition to the socioeconomic status and family dynamics, the work environment, exposure to chemicals, toxins, noise, pollution, etc., can also have a cumulative effect on the sensory system, leading to dysfunctions in the predictive coding system and to disorders like tinnitus. The characteristics of hearing loss in people exposed to continuous noise (an uninterrupted sound level that varies around less than 5 dB during the period of observation) may vary from those exposed to intermittent noise (continuous noise that persists for more than 1 s and is interrupted for more than 1 s) or impulse noise (a change of sound pressure of 40 dB or more within 0.5 s within a duration of less than 1 s) [147]. Noise-induced hearing loss is one of the major co-morbidities of tinnitus, putting construction and factory workers, musicians, soldiers and others who are exposed to either intermittent noise or impulsive noise at a higher risk of developing tinnitus. Furthermore, occupational or environmental exposure to organic solvents such as toluene, styrene, xylene and ethyl benzene can significantly impact auditory perception [148,149]. Exposure to BTEX (Benzene, Toluene, Ethylbenzene and Xylene), a cocktail of highly soluble and volatile organic compounds naturally occurring in crude oil and petroleum products, is also a major risk factor for hearing loss, as ototoxic levels of BTEX can be present in both indoor and outdoor environments [150,151,152]. Emissions from motor vehicles, petrol stations and refineries are major sources of BTEX in the outdoor environment. BTEX is estimated to constitute up to 60% of non-methane volatile organic compounds in the urban atmosphere. In the indoor environment, BTEX is found in consumer goods, such as paints and lacquers, thinners, rubber products, adhesives, inks, cosmetics and pharmaceutical products. The degree of hearing loss associated with exposure to these compounds varies with the organic solvent type and exposure level. Studies also show that patients who have an inherent sensitivity to noise and chemicals are at a higher risk of developing stress and tinnitus [153]. Per the predictive coding model, changes in the weighting of the bottom–up input are hypothesised to be one of the primary reasons that change the perceptual inference [9].

Some of the more recent events that have had a significant impact on health and economy are the COVID-19 pandemic and ongoing wars in different parts of the world. A recent study showed that 40% of a multi-country sample with tinnitus experienced exacerbated tinnitus, especially in those who were self-isolating, experiencing loneliness or sleeping poorly [154]. Furthermore, a meta-analysis of the impact of COVID-19 on cognitive health 12 weeks post-COVID showed an elevation of pro-inflammatory markers and considerable functional impairment [155]. A systematic review showed a potential relationship between pro-inflammatory markers and the development of chronic lower back pain [156].

Exposure to war is a risk factor for the development of chronic phantom perceptions owing to the exposure to loud noise and traumatic injury to the brain and other parts of the body. This relationship has been established from previous studies of war veterans [157,158]. Studies show changes in the hippocampus, anterior cingulate cortex and ventromedial prefrontal cortex which are all key regions of the predictive coding system [159]. A systematic review of the literature looking at the effect of war on children’s health reports a direct and indirect effect on their health [160]. Direct effects include a range of physical injuries affecting all bodily organs and indirect effects include a higher burden of infectious, communicable and non-communicable diseases reducing the disability-adjusted life years (DALYs) in children exposed to war. All these factors alter the signalling to the brain, likely affecting the development of the predictive coding system and resulting in maladaptive coping strategies.

### 6.3. Genetic Risk Factors

The transmission of neurological disorders through the transfer of genes is another major risk factor influencing the development of the predictive coding system and its damage later in life. From twin studies in different disorders, we infer that developmental disorders and sensory disorders such as tinnitus may be passed genetically [161,162,163]. The more interesting associations of genetic factors that may affect the health of the predictive coding system, exposing certain people more susceptible to aberrant predictive coding, are the polymorphisms of certain genes that control the signalling of specific neurotransmitter receptors.

The most prominent polymorphisms for chronic tinnitus and pain are GABA-ergic and dopaminergic receptor-related genes. Particularly, fibromyalgia is associated with the GABRB3, a GABA receptor subunit. One of the disorder-wide polymorphisms observed is that of the COMT (Val158Met) in tinnitus [164] and pain. COMT (Val158Met) is a gene that regulates the dopamine metabolism whose dysregulation is observed in all these disorders. Changes in the dopamine metabolism also affects the amplitude of the P300 component depending on the task, as explained above, potentially explaining the neural correlate of the genotype. Furthermore a genome-wide association study showed that tinnitus is related to the single-nucleotide polymorphism of *GMP6A* which is involved in the development of nuclear membranes [165]. Furthermore, both tinnitus and pain showed a shared genetic risk with other psychiatric conditions [165,166]. An array of studies also show mutations in genes affecting the cholinergic system [167] and noradrenergic system [168] in order to play a role in the generation of different neuropathologies; however, there is still a lot of variability in the results between different genetic studies. Furthermore, it is still not clear as to how these genetic risk factors affect the predictive coding system of the brain and, in turn, the development of chronic conditions.

**Figure 3:**
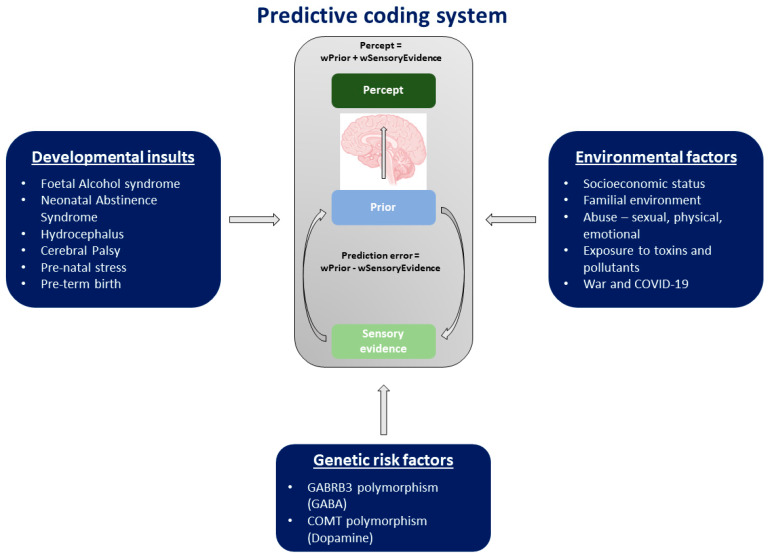
Summary of the different risk factors that can predispose a person to aberrant predictive coding. These include developmental insults and environmental and genetic risk factors.

A summary of these different risk factors is presented in Figure 3.

## 7. Limitations

The current manuscript is a perspective phrasing chronic perceptual disorders from the lens of predictive coding using a life-course approach. One of the limitations of the manuscript is not covering all the literature in this topic since this is built as a narrative review/perspective and not a systematic review. We also recognise that the predisposing factors for maladaptive changes in the predictive coding system are not limited to the ones presented here. This manuscript is built to encourage re-thinking about perceptual disorders from a predictive coding perspective and inform future research to not only probe this perspective from an experimental and systems neuroscience angle, but also consider the factors presented here from epidemiological studies and community interventions. Hence, there may be other factors which may be revealed from further prospective studies that may not be included in this manuscript and factors included in this manuscript that may not play as much of an important role as hypothesised here.

## 8. Future Directions

The current manuscript provides a basis to conduct further research in this topic of taking a life-course approach to understanding changes in the predictive coding system. From the current perspective, it is clear that there is a lot more comprehensive research performed in the field of chronic pain than tinnitus in all stages—acute, transition and chronic. Acute tinnitus is not fully understood partly because people do not see a specialist in the acute phase very quickly. Increasing awareness about tinnitus and understanding the role of acute tinnitus in terms of neural and biomarkers in the development of chronic tinnitus through longitudinal studies are the needs of the hour. The field also calls for the development of human models for tinnitus and pain to better understand the objective markers for both disorders. It is also clear that a comprehensive biopsychosocial model for tinnitus is required. This calls for epidemiological studies looking at different developmental and environmental factors for tinnitus to expand tinnitus from being an auditory problem, similar to what is being done in chronic pain. This will not only open up avenues for pharmacological and neuromodulatory interventions and the investigation of preventative factors and pre-clinical biomarkers, but also community-based, culturally appropriate interventions that can complement traditional medicine.

## 9. Conclusions

The predictive coding system is a constantly changing entity from gestation to death. In this study, we particularly studied the changes in the predictive coding system through the lens of chronic perceptual disorders. Several factors such as developmental insults and environmental and genetic factors affect different aspects of the predictive coding system influencing its development, the way associations are formed and how it processes sensory information. These factors help us identify specific groups of people who may be more vulnerable to the aberration of the predictive coding system. Future research that seeks to integrate these different factors into their questionnaires will be able to investigate biomarkers that can help us in the early detection of perceptual disorders across the life-course, thereby preventing the incidence and/or aiding in decreasing the severity of the illness which will make a significant contribution to the development of preclinical biomarkers in the predictive coding system.

## Figures and Tables

**Table 1:** Summary of findings of neural and molecular markers in the acute, chronic and transition phase of tinnitus and pain.

Phase	Neural Markers	Molecular Markers
Acute phase	Changes in auditory MMN in both tinnitus and pain. Larger P300 to deviant/novel noxious stimuli compared to standard. P300 amplitudes to novel stimuli enhanced with attention. Acute pain impacted performance on cognitive tasks Similar patterns of activation as chronic tinnitus seen in generation of acute intermittent ringing.	Increase in excitatory post-synaptic potential in acute tinnitus and pain. Pain: inflammatory “soup” that regulates pain perception. Tinnitus: changes in conductance of outer hair cells by decreasing GABA or increasing NMDA.
**Chronic Phase**: has a biopsychosocial component where the disorder is accompanied by a myriad of symptoms including reduced concentration, attention on external tasks and increased focus on the percept (pain/tinnitus), mood disorders and reduced sleep.	Pain: Changes in MMN and P300 to auditory and visual oddballs. Reduction in MMN correlates with McGill Pain Questionnaire. Increase in somatosensory ERP, possibly explained by an attentional bias towards somatosensory stimuli. Tinnitus: Mixed results in auditory oddball in the MMN timeframe. Meta-analysis showed a reduction in P300 amplitude.	Low glutamate and GABA levels in both pain and tinnitus. Pain: level of glutamate and GABA changed with different types of pain. Tinnitus: NMDA receptor activation increases tinnitus, NMDA receptor antagonist improves tinnitus.
**Transitionary phase**: psychosocial components start to play a role in this transition: Pain: systematic review shows that depression, fear avoidance, pain catastrophising, high traumatic exposure, emotional distress, feeling of helplessness and hopelessness play a role in transition from acute to chronic state. Tinnitus: Preliminary evidence suggests that depression, anxiety levels, hyperacusis, severity and frequency of hearing loss may play a role in transition from acute to chronic state.	Pain: decrease in grey matter volume in insula, nucleus accumbens, striatum and sensorimotor cortex; decreased connectivity between medial prefrontal cortex and hippocampus; connectivity between medial prefrontal cortex and nucleus accumbens determined transition accuracy by >80%. Tinnitus: Increased activity in regions of predictive coding with increased connectivity between parahippocampus and posterior cingulate cortex. Theoretical evidence suggests a change in the prediction from silence to perceiving a ringing in the absence of external stimuli.	Pain: recruitment of NMDA receptors in the dorsal horn, release of BDNF leading to a depolarising shift in dorsal horn causing GABA-related signals to become excitatory ending in hyperalgesia. Tinnitus: not a lot is known about molecular markers of transition from acute to chronic phase of tinnitus, to our knowledge.

## Data Availability

No data are included in the manuscript.
